# Dog population structure in Kumasi, Ghana: a missing link towards rabies control

**DOI:** 10.11604/pamj.2019.33.13.18284

**Published:** 2019-05-08

**Authors:** William Tasiame, Sherry Johnson, Vitus Burimuah, Ernest Akyereko, Esther Amemor

**Affiliations:** 1 School of Veterinary Medicine, Kwame Nkrumah University of Science and Technology, Kumasi, Ghana; 2 School of Veterinary Medicine, CBAS, University of Ghana, Legon, Accra, Ghana; 3 Disease Surveillance Department, Ghana Health Service, Accra, Ghana

**Keywords:** Dog population structure, rabies control, Kumasi, Ghana

## Abstract

**Introduction:**

Dog-mediated human rabies remains a major public health threat in Ghana. Dog population structure surveys are pre-requisites for appropriate planning for rabies vaccination; however, this information is unavailable in Ghana. This study describes dog population structure in Kumasi, Ghana.

**Methods:**

A household cross sectional survey was conducted from January through April 2016 in Ayeduase and Kwame Nkrumah University of Science and Technology (KNUST) campus using a structured questionnaire.

**Results:**

A total of 1319 households were surveyed out of which 35.1% (463/1319) kept dogs. We recorded 816 dogs from 463 households, giving dog to household ratio of 1.8:1. Respondents acquired 71% (579/816) of dogs through purchase. Of 2065 persons in Ayeduase, 406 owned dogs, resulting in man to dog ratio of 5.1:1. Male dogs represented 62.9% (513/816) while those aged a year and above recorded 70%. Most of the dogs were not restricted (80.3%) and 49.9% were allowed to enter neighbors' households. Dog rabies vaccination coverage was 28.1% and 64.9% in Ayeduase and KNUST campus respectively. Respondents (87.8%) from Ayeduase knew dog bite was the main means of rabies transmission, however, about 65% believed in traditional ways of treatment such as concoction, herbs and consumption of offending dogs' organs.

**Conclusion:**

The high dog to household-human ratio, increased roaming dog population and low vaccination coverage is of concern to rabies. Respondents' knowledge on their dogs is an indication of accessibility for vaccination. Obtained results can be useful for rabies vaccination planning in Kumasi and other comparable settings in Ghana.

## Introduction

Rabies is among the deadliest zoonotic diseases in the world, with an estimated case fatality rate of almost 100%. Human rabies can be controlled through elimination of the disease in animals. Rabies deaths in human are in the range of 70,000s each year. Rabies tops annual human deaths from all zoonoses and is likely number 1 vaccine preventable disease that kills most [1]. The disease affects poor-rural communities across most parts of Africa and Asia [2]. Rabies in the domestic dog has been on the increase in most parts of sub-Saharan Africa due to low vaccination coverage [3]. Thousands of lives are lost each year due to rabies mostly in children. Reported data on rabies is considered under-reported. This appears to be one of the reasons for low priority in commitment and resource allocation for control and elimination of rabies [4]. In Ghana, rabies incidence has been on the increase since the government stopped funding free mass vaccination in 1997 [5]. Afakye further noted human rabies cases are clinically diagnosed in terminal stages without laboratory confirmation while reported animal rabies to the veterinary services is mostly low. Even when these cases are reported, little or no investigation is often conducted. Ghana has three zonal veterinary and five regional laboratories which are not well equipped. According to the World Organisation for Animal Health (OIE) performance veterinary services gap report [6], there is the need to strengthen passive surveillance for zoonotic diseases such as rabies, tuberculosis, brucellosis and cisticercosis. Same report indicated a yearly target of 15,000 dogs and cats to be vaccinated against rabies in main cities of Ghana as advised by the public health division of veterinary services. It is obvious this number may not curtail the menace of rabies which is known to affect dogs and humans mainly in the rural areas of developing countries like Ghana. Knowledge and understanding of population density, structure and ownership of dogs is vital to the planning and execution of mass vaccination against rabies in dogs in a given area [7, 8]. Cultural practices determine the level of supervision of the social interactions of dogs and access to resources (food, water, shelter and mates). It is assumed that high-density dog populations permit the occurrence of enzootic canine rabies; but this is not very well documented [9]. Studies have shown positive correlation between dog and human population [10] as represented in a 30% increase in Ghana's human population from 2000 to 2010 [11]. Dog ecology studies are directly linked to the epidemiology of dog, thus a better understanding of this linkage is crucial to the design and planning of effective rabies control measures [12].

Knowledge about the size and turnover of the dog population concerned, degree of supervision, owned and unowned population, accessibility and socio-cultural attitudes of community members are all crucial and dependent on the success of rabies control measures. Where the number of dogs is not registered by licensing, questionnaire surveys can fill this gap through street dog counts and household interviews [13]. High numbers are reported of the domestic dog and it is estimated to be over 700 million [14]. In Chile there was high proportion of households owning dogs in rural areas (89%) than in towns (63%) or cities (49%). Population growth was as high as 20% in cities, 19% in towns and 9% in rural areas. The three different settings allowed dogs to roam freely [15]. A study in Tanzania reported 13% of all households owned dogs. There was high fertility with 5.5 pups per litter. Another observation was dog population recording 6 times higher than assumed by town records. The interesting part was the high birth and death rates which mean high turnover rate and decline in immunity if vaccination is not repeated every year to capture new dogs [16]. In Niger state, Nigeria, there was an increase in dog to human ratio from 1:12.1 in 1999 to 1:5.4 in 2017 indicating a closer interaction between dogs and humans which could pose public health outcomes. About 55% of dogs roamed freely to other people's homes at night for food and mating [17]. A 56 minute hotline documentary captioned “Deadly Pets” Ghana shook the minds of many citizens as rabies became a topic of discussion. Sad pictures of human and animal cases coupled with emotional accounts from relatives mostly from less endowed Ghanaian communities in Kumasi and its environs proved rabies to be a public health menace in Ghana [18]. In a single rabies outbreak in the upper east region of Ghana, nine human lives were lost [19]. Community dogs were responsible for these bites. Similarly, a stray dog was associated in the first reported case of dog-associated pig rabies in Ghana [20]. A presumptive case of clinical human rabies that survived in Ghana without administration of post exposure prophylaxis after the dog that bit this patient tested positive of rabies virus calls for concern and further investigation [21]. A search of literature revealed no published paper from Ghana on dog population studies and the role of community members in the perpetuation and control of rabies. The known papers from Ghana focused on secondary data on rabies which stated endemicity in Accra the capital city of Ghana [22]. Another study found 278 persons died of rabies in a period of 13 years through morbidity and mortality data obtained from Medical Statistics Divisions in the Ministry of Health, Ghana [23]. Considering the above stated paucity of information on the subject and the need to provide evidence which is likely to attract the attention of local and international organizations, it becomes necessary to elucidate dog population structure in parts of Kumasi in Ashanti region of Ghana, a data which can assist in planning dog antirabies vaccination campaigns.

## Methods

**Figure 1:**
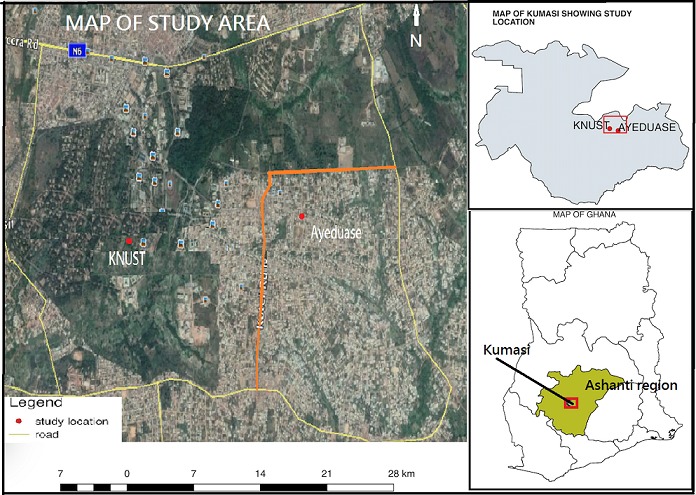
Map of the study sites at Ayeduase

**Study site:** the study was carried out at Ayeduase north and south and Kwame Nkrumah University of Science and Technology (KNUST) campus (Figure 1). Ayeduase is one community but was divided into North and South for the purpose of this study. Ayeduase and KNUST belong to Oforikrom sub metro of Kumasi Metropolitan Assembly in Ashanti region. Ayeduase lies between latitude 6.67 and longitude -1.54 with an average temperature of about 25.67^o^C while KNUST is located between latitude 6.6747 to longitude -1.5717. The human population of Ayeduase is estimated at 29,748 according to the 2012 population census and that of KNUST is estimated to be 40,000 persons [24]. The senior staff residential area consists of 30 streets with 383 households. They are highly educated persons mostly lecturers of the university and are financially sound. Junior staff residential area is made up of 7 different areas of 124 units with 192 households. These are labourers with little or no formal education. KNUST campus was selected due to visible free roaming dogs on campus and its possible risk to such a high number of persons. Ayeduase is becoming a cosmopolitan community due to its closeness to the university with business opportunities.

**Figure 2:**
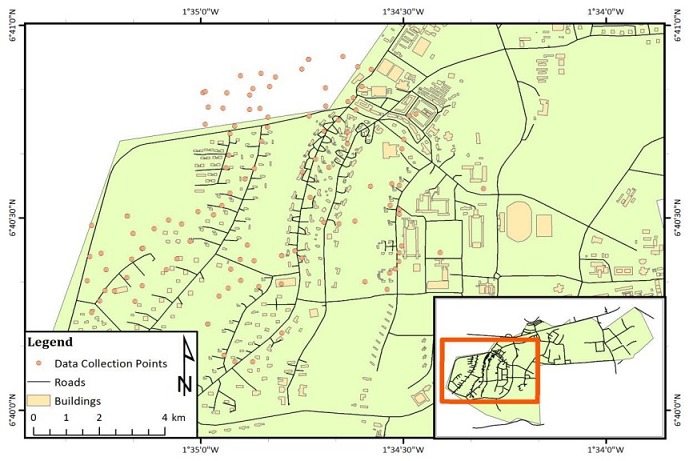
Data collection points in KNUST

**Study design and data collection method:** a cross sectional study was carried out to determine dog population structure within the months of January through April 2016 at Ayeduase and KNUST campus. All the households on the 30 streets at the senior staff area and the 7 different areas of the junior staff on KNUST campus (Figure 2) were sampled. The households without dogs were not included in the data collection. Five streets each were randomly selected at Ayeduase north and south and every third household was surveyed until the end of the street. The agreement was to survey not less than 500 households to meet the WHO guidelines for dog density survey which recommends sampling 500-5000 households [13]. A two part structured questionnaire was administered to the head or senior member of each household. Part A of solicited demographic information of owners, dog ownership, bite history and knowledge on rabies. Part B sought specific information on individual dogs such as sex, age, antirabies vaccination history, feeding habits, movement and confinement. In cases that no member of a household was sufficiently literate, the questions were read out and explained to the respondent in the local language and the answers given were filled in by the interviewer. The rationale behind this study was explained to all potential respondents who were at liberty to participate or decline. Data was analysed in Microsoft Excel for percentages. Below are the map of the study site at Ayeduase (Figure 1) and the map of the data collection points in KNUST (Figure 2).

## Results

A total of 1319 households were surveyed out of which an average of 35% owned dogs. The representation of KNUST campus in the study was 44% (575/1319) with 40.5% of households owning dogs. Ayeduase north recorded a total of 278 dogs from 138 households which suggests a ratio of 2 dogs in each household (2:1). Similarly, Ayeduase south and KNUST had more than a dog in each household giving an average dog to household ratio in the 3 sites as 1.8:1. Out of the 230 households from Ayeduase north and south, we recorded a total of 2065 persons which gave an average of 9 persons per household. Dog to man ratio was 1:5.1 (2065/406) from Ayeduase as shown in Table 1. There was a male dominance of 50% (408/816), 33.9% of females and a 3.2% of altered females as observed in the 3 sampling sites. Younger dogs aged less than a year represented 30% with the remaining 70% of dogs in the reproductive age of a year and above shown as 1-2 years (32.5%), 3 years (18.9%), 4 years (7%) and above 5 years (11.6%). The survey revealed that out of the 816 dogs owned, a 71% (579/816) were purchased while the rest were either obtained as gift or from own litter. Ayeduase north had a low percentage of dog acquisition as gift (1.4) followed by Ayeduase south (14.4) but interestingly, households on KNUST campus acquired through gifts more than 6 times what Ayeduase households obtained as shown in Table 2. Most of the dogs from Ayeduase were left to scavenge representing almost 50%, similarly, some 45.8% were fed with left overs or food consumed by the families in these households while a small number of 20 dogs (4.9%) benefited from commercially prepared feed. Furthermore, a high percentage of dogs from all the 3 sample sites were left to roam without restriction (80.3%) leaving just 91 (19.7%) dogs that were kept home in kennels or chains. There was virtually no difference on dogs allowed to enter other households that do not belong to their owners for feeding or playing recording almost 50% each. The main method of depopulation of dogs in Ayeduase was through sale to interested buyers (69.6%) but an interesting 5.6% of dogs were said to be slaughtered for human consumption. The study showed low vaccination rates of 28.1% in Ayeduase but that of KNUST was as high as 65% which assisted in the average rate of 46.6%. A high 53.4% of dogs were not vaccinated against rabies in the selected study communities. These vaccinations were conducted by the veterinarian (60.5%) but 13.2% was done by the owners or their friends. Dog bite incidence recorded 3.9% of the 463 dog owning households sampled. Half of these bites were from owned dogs and the other half from neighbor's dogs (Table 3). Dog owning household respondents from Ayeduase had different views on rabies transmission and treatment. Majority considered dog bite (87.8%) as the main source of rabies transmission and the rest either had no idea or thought playing with dog and scratches could also be other sources to acquire the disease. Some 35.2% of households said treatment against bites from suspected rabid dogs could only be received at the hospital and were not aware of any traditional means of treatment. However, a great value of 64.8% considered traditional means such as concoction (29.1%), herbs (26.5%) and consumption of offending dogs' organs as the medium of treatment when bitten by suspected rabid dogs (Table 4).

**Table 1:** Dog and human population density in Ayeduase and KNUST campus

Variables	Frequency			Total No. (%)
	Ayeduase North	Ayeduase South	KNUST campus	
No. of households sampled	420	324	575	1319
No. households with dogs	138 (32.9)	92 (28.4)	233 (40.5)	463(35.1)
Total number of dogs	278	128	410	816
Dog to household ratio	2:1	1.4:1	1.8:1	1.8:1
No. of persons in households with dogs	1258	807	-	2,065
No. of persons per household	9.1	8.8	-	9
Dog to man ratio	1:4.5	1:6.3	-	1:5.1

Key: %= percentage, - = no data, No. = Number

**Table 2:** Dog population structure in Ayeduase and KNUST campus

Variables	Frequency			Total No. (%)
	Ayeduase North	Ayeduase South	KNUST campus	
**Sex**				
Male	143 (51.4)	55 (42.9)	210 (51.2)	408 (50)
Female	86 (31)	49 (38.3)	142 (34.6)	277 (33.9)
Altered male	32 (11.5)	18 (14.1)	55 (13.4)	105 (12.9)
Altered female	17 (6.1)	6 (4.7)	3 (0.7)	26 (3.2)
Total	278	128	410	816 (100)
**Age**				
<1 year	75 (27)	36 (28.1)	134 (32.7)	245 (30)
1-2 years	97 (34.9)	42 (32.8)	126 (30.7)	265 (32.5)
3 years	56 (20.1)	26 (20.3)	72 (17.5)	154 (18.9)
4 years	17 (6.1)	9 (7.0)	31 (7.6)	57 (7)
5 years & older	33 (11.9)	15 (11.7)	47 (11.5)	95 (11.6)
Total	278	128	410	816 (100)
**Source of dog**				
Bought	246 (88.5)	104 (81.2)	229 (55.9)	579 (71)
Gift	4 (1.4)	18 (14.1)	141 (34.4)	163 (19.9)
Own litter	28 (10.1)	6 (4.7)	40 (9.7)	74 (9.1)
Total	278	128	410	816 (100)

Key: %= percentage, No. = Number

**Table 3:** Management and care for dogs in Ayeduase and KNUST campus

Variables	Frequency			Total No. (%)
	Ayeduase North	Ayeduase South	KNUST campus	
				
**Feeding of dogs**				
Fed on commercial feed	7 (2.5)	13 (10.2)		20 (4.9)
Fed on family left over	138 (49.6)	48 (37.5)	-	186 (45.8)
Scavenge	133 (47.8)	67 (52.3)		200 (49.3)
Total	278	128		406 (100)
**Movement control**				
Restricted at home	19 (13.8)	14 (15.2)	58 (24.9)	91 (19.7)
No restriction	119 (86.2)	78 (84.8)	175 (75.1)	372 (80.3)
Total	138	92	233	463 (100)
**Do you allow other dogs enter your household**				
Yes	112 (81.2)	18 (19.6)	101 (43.3)	231 (49.9)
No	26 (18.8)	74 (80.4)	132 (56.7)	232 (50.1)
Total	138	92	233	463 (100)
**Depopulation method**				
Sell off	88 (63.8)	72 (78.3)		160 (69.6)
Give as gift	37 (26.8)	17 (18.5)		54 (23.5)
Slaughter for consumption	10 (7.2)	3 (3.2)	-	13 (5.6)
Euthanasia	3 (2.2)	0 (0)		3 (1.3)
Total	138	92		230 (100)
**Vaccination status**				
Yes	82 (29.5)	32 (25)	266 (64.9)	380 (46.6)
No	196 (70.5)	96 (75)	144 (35.1)	436 (53.4)
Total	278	128	410	816 (100)
**Who vaccinated dog**				
Veterinarian	75 (91.5)	23 (71.9)	132 (49.6)	230 (60.5)
Animal health technician	4 (4.9)	5 (15.6)	91 (34.2)	100 (26.3)
Self or friend	3 (3.6)	4 (12.5)	43 (16.2)	50 (13.2)
Total	82	32	266	380 (100)
**Dog bite incidence**				
Yes	4 (2.9)	3 (3.3)	11 (4.7)	18 (3.9)
No	134 (97.1)	89 (96.7)	222 (95.3)	445 (96.1)
Total	138	92	233	463 (100)
**Whose dog**				
Own dog	0	0	9	9 (50)
Neighbor’s dog	4	3	2	9 (50)

Key: %= percentage, - = No data, No. = Number

**Table 4:** Knowledge of respondents on rabies in Ayeduase

Variables	Frequency		Total No. (%)
	Ayeduase North	Ayeduase South	
**Means of rabies transmission**			
Dog bite	126 (91.3)	76 (82.6)	202 (87.8)
Playing with dog	1 (0.7)	1 (1.1)	2 (0.9)
Scratch, bite & playing with dog	1 (0.7)	3 (3.3)	4 (1.7)
Don’t know	10 (7.2)	12 (13)	22 (9.6)
Total	138	92	230 (100)
**Traditional ways of treating rabies**			
Concoction	56 (40.6)	11 (12)	67 (29.1)
Herbs	27 (19.5)	34 (37)	61 (26.5)
Consumption of offending dog organ	16 (11.6)	5 (5.4)	21 (9.1)
Don’t know i.e. only aware of hospital	39 (28.3)	42 (45.6)	81 (35.2)
Total	138	92	230 (100)

Key: %= percentage, No. = Number

## Discussion

The aim of this study was to determine dog population structure and knowledge gaps that may favor dog-mediated human rabies in parts of Kumasi in Ashanti region which is the most populous region in Ghana. According to existing literature this is the first demographical data of dogs in Ghana that can assist in appropriate design of rabies control strategies. These are likely to assist vaccination planning in other Ghanaian community settings similar to the study area taking into consideration the expensive and time consuming nature of dog population census. Dog density of 35.1% from households surveyed in this study is relatively high compared to other studies from Uganda (12.9%) [25], Tanzania (13.3%) [16], South Africa (17%) [26] and Nigeria (24.9%) [27] respectively. A study in Namibia with a limited number of 245 participants aged 18-64 years obtained 65.5% of household dog ownership [28]. Similarly, 87% dog owning households emerged in a 390 household interview in Kenya [29]. Our study also recorded an average of 2 dogs in a household (dog to household ratio=1.8:1). Furthermore, we got an average of 9 persons in each household and a dog to man ratio of 1:5.1 from Ayeduase. These results are obviously high indicating closer interaction between dogs and humans with possible repercussion of zoonoses mainly rabies if these dogs do not receive appropriate veterinary care and in particular protection against rabies. Intact male dogs dominated in this study recording 50% while females showed 33.9%. This result runs through several studies with 61.2% from Antanarivo, Madagascar [12] and 62.2% from Nigeria [7]. Sterilization of both males and females was low recording 12.9% and 3.2% respectively. The study from Anne Conan cited earlier indicated a low 1-1.5% of dogs were castrated. This could be as a result of unwillingness from dog owners for want of puppies or probably due to low veterinary presence and high cost of such surgical interventions especially in the case of females. About 30% of dogs were below a year old leaving a high 70% of dogs in their reproductive stage which may lead to frequent reproduction and increase in dog population with its dependence on the human population already burdened with other financial obligations. The Antanarivo survey also recorded a 38.8% of dogs below a year old. It was interesting to note that majority (71%) of the dogs were sourced through purchase and as little as 9.1% remained with the owners from own litter. This result was confirmed in the mode of depopulation where similar figure (69.1%) of the dogs was sold. This may mean dog owners value them so much to spend money to purchase them and probably it is also a business venture as they sell almost 70% of these dogs out for financial gain. A figure of 5.6% of dogs was slaughtered for human consumption. It is known that some tribes from the northern and eastern parts of Ghana consume dogs so this could be the reason for this result since Ayeduase harbors people from different regions across Ghana due to its thriving business opportunities provided by the university. Despite the plausible evidence of importance given by owners to dogs through purchase and sale, this does not reflect in the mode of feeding. Almost half (49.3%) of these dogs in Ayeduase are left to scavenge and a 45.8% are fed on family left over food leaving only 4.9% being fed on commercially prepared feed which may be the ideal for their maintenance and proper growth.

Some risk factors of rabies were equally assessed in this study. Most of the dogs (80.3%) in Ayeduase and KNUST campus were left to roam leaving only 19.7% restricted at home in kennels or chains. More so, almost half (49.9%) of these dogs are said to be allowed to enter other households for food, water and probably playing with dogs from these homes. In addition, only 46.6% of dog rabies vaccination coverage was achieved which is below the 70% WHO recommendation [13]. Vaccination figure from KNUST campus was 64.9% while that from Ayeduase recorded only 28.1% indicating a great disparity probably due to the difference in educational levels favoring staff of a university institution. The average vaccination coverage (46.6%) is higher than that obtained (21.7%) by [27] and 33% [26] but similar to 47.9% [7]. Reasons favoring low canine rabies vaccination coverage in Africa are listed as: (a) a low priority given for disease control as a result of lack of awareness of the rabies burden; (b) epidemiological constraints such as uncertainties about the required levels of vaccination coverage and the possibility of sustained cycles of infection in wildlife; (c) operational constraints including accessibility of dogs for vaccination and insufficient knowledge of dog population sizes for planning of vaccination campaigns; and (d) limited resources for implementation of rabies surveillance and control [30]. The high roaming dog population coupled with low protection of dogs against rabies is of serious concern for emergence of rabies in these communities. These parameters put humans, dogs and other animals at high risk if nothing is done in preparedness towards an outbreak of rabies and other zoonotic diseases transmitted by dogs. Notwithstanding these figures, dog bite incidence was 3.9% of which half came from owned dogs and the other half from neighbors' dogs. Even though this figure appears low, one should still be worried if a single rabid dog bites a person without post exposure prophylaxis since this can be fatal resulting in loss of human life. A single death through a preventable disease as rabies is one too much to record. Majority (87.8%) of persons interviewed had good knowledge about the means of transmission of rabies, picking dog bite as the right answer. This may be because rabies cases have been mentioned in the region frequently through the print and airwaves from human rabies deaths in Ashanti region. The study from Kenya [29] recorded similar result of 79% of dog bite as the main source of rabies transmission but in Ethiopia about 40% had misunderstanding believing spirits cause the disease [31]. When asked about traditional means of rabies treatment, only 35.2% of our participants said the only means of rabies treatment takes place at the hospital. The rest of 64.8% believe the use of concoction, herbs and consumption of offending dogs' organs as a treatment remedy for rabies. Similarly, respondents from same Ethiopian study said traditional means of treatment is the best cure for rabies. The believe in traditional methods of treatment may be inimical to the fight against human rabies since these are not backed scientifically and are likely to fail with dying consequences.

## Conclusion

There was high dog to household and dog to human ratio in the study sites. The close interaction of dogs and humans is of concern to rabies and other dog related zoonoses. Dog ownership may be considered as a business venture in view of the high purchase and sale yet low attention is given to these dogs in Ayeduase and KNUST campus. Despite the low restriction favoring roaming dogs in the study sites, knowledge of respondents of their dogs is an indication of accessibility of these dogs for vaccination. Traditional means of rabies treatment such as use of concoction, herbs and consumption of offending dogs’ organs may cause more human deaths if unchecked. It is believed that this first time preliminary data on dog population structure may fill that missing link providing data for organizations ready to assist in rabies vaccination through effective planning and execution of campaigns. We recommend an upscale of such studies in other parts of Ghana which may be handy to organizations working towards the elimination of rabies in Africa.

### What is known about this topic

- Rabies is a major public health threat in Africa;
- The domestic dog is the main source of rabies transmission in developing countries;
- Dog population studies offer requisite information for vaccination programs.

### What this study adds

- Dog to household ratio is high in the study area;
- Coverage of dog antirabies vaccination was low;
- Dog owners still believe in superstition for rabies treatment.

## Competing interests

The authors declare no competing interests.
